# Supplementary material for: Production in Bacteria and Characterization of Engineered Humanized Fab Fragment against the Nodal Protein
Source: Pharmaceuticals (Basel). 2023 Aug 10;16(8):1130. doi: 10.3390/ph16081130 (PMC10459755; doi:10.3390/ph16081130)
Supplement: Supplementary file 1 [file pharmaceuticals-16-01130-s001.zip › pharmaceuticals-2504366-supplementary.pdf]

SUPPLEMENTARY MATERIALS

Production in Bacteria and Characterization of Engineered Humanized Fab Fragment against the Nodal Protein

Jwala P. Sivaccumar [1,†], Emanuela Iaccarino [1], Angela Oliver [1,2], Maria Cantile [3], Pierpaolo Olimpieri [4], Antonio Leonardi [5], Menotti Ruvo [1,*] and Annamaria Sandomenico [1,*]

1 Institute of Biostructures and Bioimaging, CNR, Via P. Castellino, 111, 80131 Naples, Italy; jwala.priyadarsini@gmail.com (J.P.S.); emanuela.iaccarino@gmail.com (E.I.); oliver.angelaa08@gmail.com (A.O.)
2 Università degli Studi della Campania Luigi Vanvitelli, Via Vivaldi 43, 81100 Caserta, Italy
3 BIOVIIIX, via A. Manzoni, 1, 80123 Naples, Italy; maria.cantile@bioviiix.com
4 Department of Physics, Sapienza University, 00184 Rome, Italy; pierpaolo.olimpieri@gmail.com
5 Department of Molecular Medicine and Medical Biotechnologies, University of Naples "Federico II", via Pansini 5, 80131 Naples, Italy; leonardi@unina.it
* Correspondence: menotti.ruvo@unina.it (M.R.); annamaria.sandomenico@cnr.it (A.S.)
† Present address: Texas Therapeutics Institute, Brown Foundation Institute of Molecular Medicine, McGovern Medical School, The University of Texas Health Science Center at Houston, Houston, TX 77030, USA.

Citation: Sivaccumar, J.P.; Iaccarino, E.; Oliver, A.; Cantile, M.; Olimpieri, P.; Leonardi, A.; Ruvo, M.; Sandomenico, A. Production in Bacteria and Characterization of Engineering Humanized Fab Fragment against the Nodal Protein. *Pharmaceuticals* **2023**, *16*, 1130. https://doi.org/10.3390/ph16081130

Academic Editor: Gill Diamond

Received: 29 June 2023
Revised: 29 July 2023
Accepted: 2 August 2023
Published: 10 August 2023

Copyright: © 2023 by the authors. Licensee MDPI, Basel, Switzerland. This article is an open access article distributed under the terms and conditions of the Creative Commons Attribution (CC BY) license (https://creativecommons.org/licenses/by/4.0/).

Light chain (220 residues)

DIQMTQSPSSLSASVGDRVTITCKSSQSLLNSGNQKNYLTWYQQKPGKAPKLLIYWASTR
ESGVPSRFSGSRSGTDFTLTISSLQPEDFATYYCQNDYSYPLTFGQGTKVEIKRTVAAPSV
FIFPPSDEQLKSGTASVVCLLNNFYPREAKVQWKVDNALQSGNSQESVTEQDSKDSTYS
LSSTLTLSKADYEKHKVYACEVTHQGLSSPVTKSFNRGEC

Heavy chain (239 residues)

EVQLVESGGGLVQPGGSLRLSCAASGFTFRNYWMSWVRQAPGKGLEWVAEIRLKSDNY
AARYAESVKGRFTISRDTSKNTAYLQMNSLRAEDTAVYYCSGIRRFAYWGQGTLVTVSSA
STKGPSVFPLAPSSKSTSGGTAALGCLVKDYFPEPVTVSWNSGALTSGVHTFPAVLQSSG
LYSLSSVVTVPSSSLGTQTYICNVNHKPSNTKVDKKVEPKSCDKTHTGSGALQPTQGAMP
A

Figure S1. Sequence of light and heavy chains of recombinant humanized Fab 3D1. The variable domain (black), the constant domain (red) and the MTG site (blue) are highlighted.

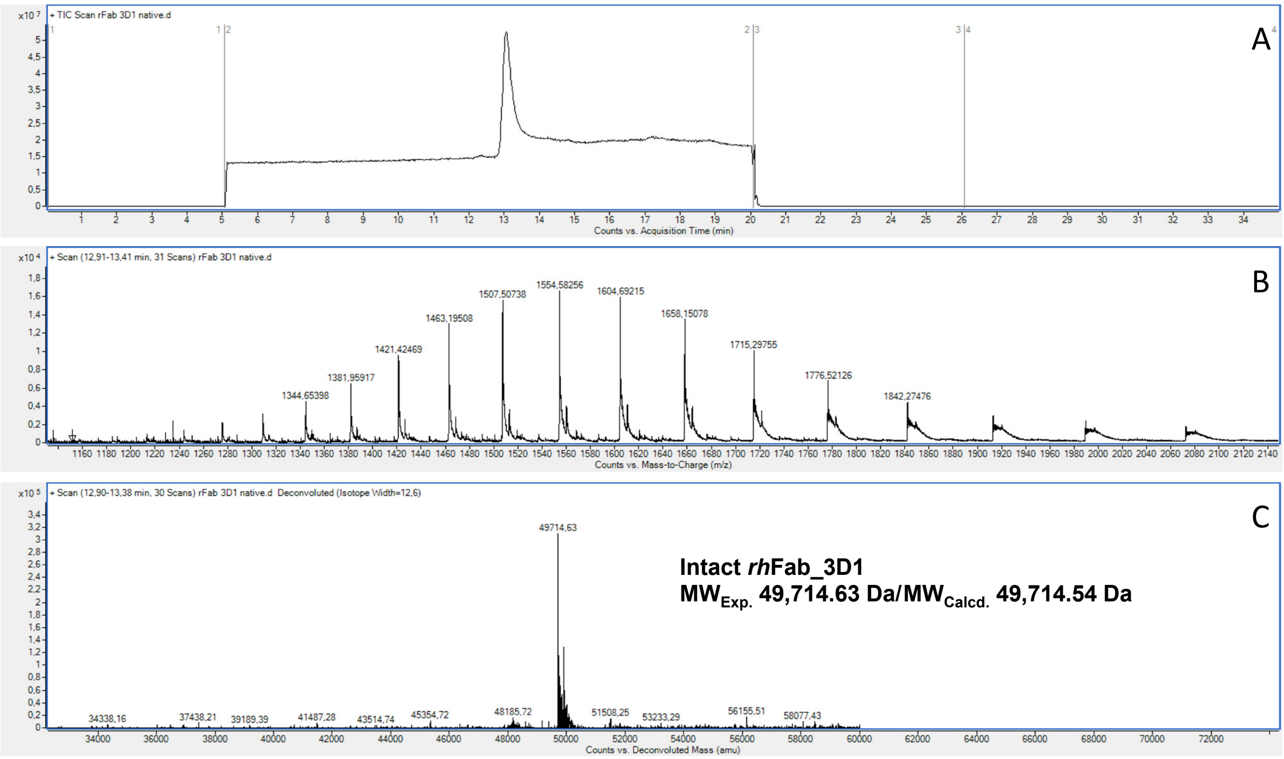

Figure S2. LC-ESI-TOF-MS analysis of the intact *rh*Fab_3D1 including the TIC chromatogram (A), the multicharged spectrum (B) and the deconvoluted mass spectrum (C). The calculated molecular weight is also reported on Figure S2C. $MW_{Exp.}$ 49714.54 Da/$MW_{Calcd.}$ 49714.63 Da.

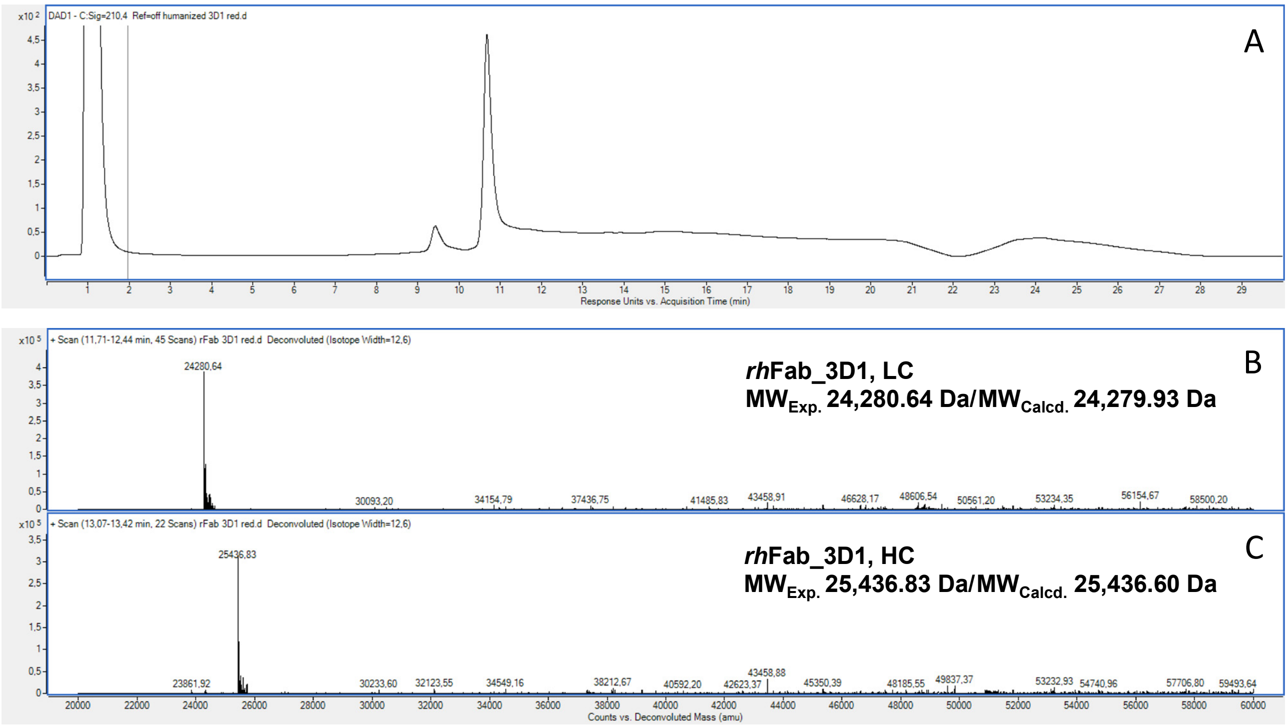

Figure S3. LC-ESI-TOF-MS analyses of the *rh*Fab_3D1 under reducing conditions, including the UV chromatogram at 210 nm (A) in which the first peak corresponds to the light chain while the second peak to the heavy chain. In (B) and (C) the deconvoluted mass spectra of the heavy and light chains are reported, respectively

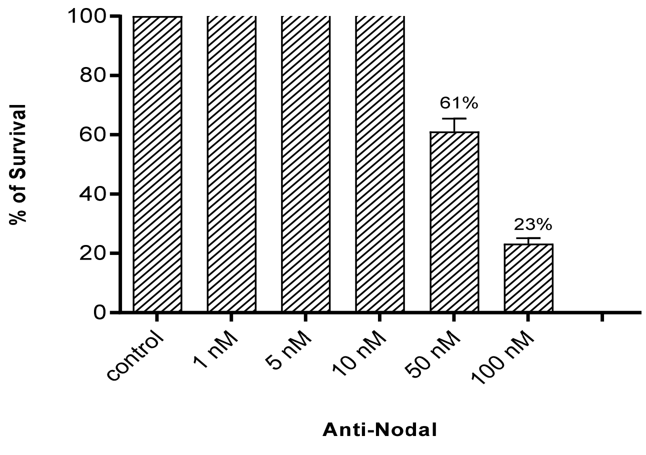

Figure S4. *In vitro* NT2-D1 cell growth inhibition assays performed with the commercial anti-Nodal WS65 antibody at concentrations ranging between 1 nM and 100 nM. The data account for the average of three independent experiments performed on triplicate wells for each data point.
